# Heterogeneous Anion-Exchange Membranes with Enhanced Ion Conductivity for Continuous Electrodeionization

**DOI:** 10.3390/membranes13120888

**Published:** 2023-11-27

**Authors:** Ji-Min Lee, Moon-Sung Kang

**Affiliations:** Department of Green Chemical Engineering, College of Engineering, Sangmyung University, Cheonan 31066, Republic of Korea; 2023D3006@sangmyung.kr

**Keywords:** heterogeneous anion-exchange membrane, ionomer binder, continuous electrodeionization, poly(2,6-dimethyl-1,4-phenylene oxide), ion-exchange capacity, nanofiber powder

## Abstract

In this study, the optimal fabrication parameters of a heterogeneous anion-exchange membrane (AEM) using an ionomer binder are investigated to improve the performance of continuous electrodeionization (CEDI) for producing ultrapure water. Poly(2,6-dimethyl-1,4-phenylene oxide) (PPO) is selected as the base material for preparing the ionomer binder and quaternized to have various ion exchange capacities (IECs). The optimal content of ion-exchange resin (IER) powder according to the IEC of the ionomer binder is then determined through systematic analyses. In conclusion, it is revealed that a heterogeneous AEM with optimal performance can be fabricated when the IEC of the ionomer binder is lowered and the content of IER powder is also lower than that of conventional heterogeneous membranes. Moreover, crosslinked quaternized PPO (QPPO) nanofiber powder is used as an additive to improve ion conductivity without deteriorating the mechanical properties of the membrane. The membrane fabricated under optimal conditions exhibits significantly lower electrical resistance (4.6 Ω cm^2^) despite a low IER content (30 wt%) compared to the commercial membrane (IONAC MA-3475, 13.6 Ω cm^2^) while also demonstrating moderate tensile strength (9.7 MPa) and a high transport number (ca. 0.97). Furthermore, it is proven that the prepared membrane exhibits a superior ion removal rate (99.86%) and lower energy consumption (0.35 kWh) compared to the commercial membrane (99.76% and 0.4 kWh, respectively) in CEDI experiments.

## 1. Introduction

Continuous electrodeionization (CEDI) is a hybrid separation process that combines electrodialysis (ED) and ion exchange processes for producing ultrapure water [1,2,3]. The CEDI has the special feature of maintaining the ion conductivity of the solution, even at low concentrations, by filling compartments with ion-exchange resin (IER or IX resin). Since IER is continuously regenerated through water dissociation during operation, it can be environmentally friendly and economically advantageous compared to conventional ion exchange processes that require laborious chemical regeneration [2,3].

Meanwhile, ion-exchange membranes (IEMs) are key materials that determine the performance of electro-membrane processes such as CEDI [4,5,6,7], electrodialysis [4,8,9,10], reverse electrodialysis [11,12,13,14], Donnan dialysis [5,15], and fuel cells [5,13]. IEMs can be classified into anion-exchange membranes (AEMs), cation-exchange membranes (CEMs), and bipolar membranes (BPMs) depending on the types of fixed ion groups they contain [16,17]. IEMs are also divided into a homogeneous type, made solely of polymers attaching fixed charge groups, and a heterogeneous type, composed of ion-conductive resin powder and inert polymer binder [5,8,10,15]. Homogeneous IEMs offer the advantages of low electrical resistance and high permselectivity, but they also have disadvantages, including low mechanical strength and high production costs [8,10,15]. In contrast, heterogeneous IEMs are cost-effective to produce but face challenges such as relatively high electrical resistance (low ion conductivity) and reduced permselectivity due to the intermittent connectivity of the conductive regions [10,18,19]. Despite their inferior performance compared to homogeneous membranes, heterogeneous IEMs have found extensive use in various electrochemical systems, owing to their simple manufacturing process and cost-effectiveness [8,10,17]. Additionally, it has been reported that the heterogeneity of membrane surfaces promotes the onset and growth of electroconvection, enhances ion transport, and reduces fouling, among other benefits. Consequently, there is considerable potential for improving the performance of heterogeneous membranes [18,19].

The ion conductivity of a heterogeneous IEM is contingent upon both the content of IER particles within the membrane and the bulk and surface morphology of the membrane [19,20,21]. Notably, the quantity of resin powder used during the production of a heterogeneous membrane profoundly influences the ion exchange capacity (IEC) and water content of the membrane, consequently wielding substantial influence over its overall electrochemical characteristics [17]. Meanwhile, inert polymers are typically utilized as binding agents to stabilize resin powder particles, resulting in membranes with a low swelling ratio and excellent mechanical properties. When fabricating a heterogeneous IEM, the content of IER powder is typically 50 to 70 wt%. If the content exceeds this range, the mechanical strength and shape stability of the membrane may be compromised [20,22]. Additionally, IEMs with a high resin powder content tend to have a higher swelling ratio in water, which can reduce the permselectivity of the membranes [20]. Therefore, determining both the content of resin powder and the type of binder is a critical factor in producing high-performance heterogeneous IEMs [15,17,22].

Several studies have aimed to enhance the performance of heterogeneous IEMs through binder modifications. Incorporating hydrophilic polymers like polyethylene glycol (PEG) has improved membrane hydrophilicity and ion transfer efficiency. However, this led to increased water content, resulting in reduced mechanical strength and permselectivity [23,24,25,26]. Meanwhile, Wang et al. utilized sulfonated polystyrene microspheres in heterogeneous CEMs to enhance ion transport [27]. Miao et al. fabricated heterogeneous AEMs using *N,N*-dimethyl aminoethyl methacrylate to improve crosslinking [22], but faced challenges with high electrical resistance. Mubita et al. developed heterogeneous AEMs using an ionomer binder, achieving an IEC close to that of homogeneous membranes, and found a correlation between nitrate selectivity and the hydrophobicity of alkyl groups [26]. Nonetheless, the high water content of these membranes (over 40%) could potentially degrade their mechanical properties.

**Figure 1:**
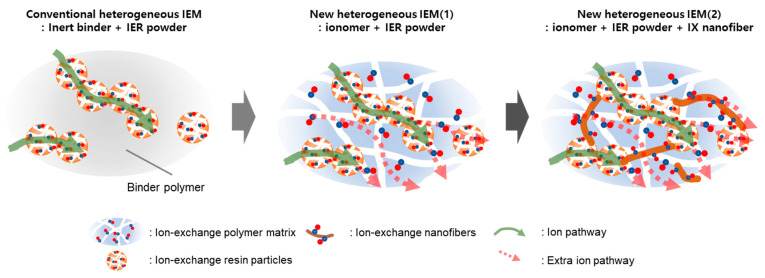
A schematic diagram showing the main idea of this study.

In this work, heterogeneous AEMs were fabricated using a charged polymer as a binder. While previous studies have explored the use of ionomers as binders, investigations focused on establishing optimal fabrication conditions—specifically balancing the IEC of the binder and the resin powder content—have not yet been conducted. Furthermore, the ionomer binder used in prior research typically has a high IEC, comparable to that of homogeneous membranes, and a substantial water content. This presents the disadvantage of potentially deteriorating the physical properties of the membrane. Therefore, we attempted to determine the optimal conditions for the ionomer binder and the content of IER powder with an ideal IEC by comprehensively considering membrane properties such as mechanical strength and electrical resistance. The ionomer binder was prepared by introducing quaternary ammonium groups into poly(2,6-dimethyl-1,4-phenylene oxide) (PPO). First, PPO was modified into brominated PPO (BPPO) through a bromination reaction. Subsequently, it was converted into quaternized PPO (QPPO) with various IECs by adjusting the amount of trimethylamine (TMA) added. We determined the optimal fabrication conditions for heterogeneous membranes through a systematic performance evaluation of membranes prepared using ionomer binders with varying IECs. Furthermore, to enhance the connectivity between IER particles and improve ionic conductivity without compromising mechanical strength, crosslinked QPPO nanofiber (IX nanofiber) was introduced as an additive to the membranes. The main idea of this study is illustrated in Figure 1. The performance of the heterogeneous membrane fabricated under these optimal conditions was then evaluated by applying it to a CEDI process for producing ultrapure water.

## 2. Materials and Methods

### 2.1. Materials

PPO (*M_w_* = 30,000 g/mol) [28], used as the base polymer for preparing the ionomer binder, was purchased from Sigma-Aldrich (St. Louis, MO, USA). It was treated with bromine, sourced from Daejung Chem. Ind. (Siheung, Republic of Korea), to produce BPPO. For solvents, chlorobenzene was used for PPO and 1-methyl-2-pyrrolidone (NMP) for BPPO. Trimethylamine (TMA) served as the reagent for the quaternization of PPO, while *N*,*N*,*N*′,*N*′-tetramethylethylenediamine (TMEDA) was used for crosslinking QPPO. All these reagents were acquired from Sigma-Aldrich (USA) and were used without further purification. The IER powder, necessary for producing the heterogeneous membranes, was obtained by pulverizing a commercial IER (LPF860, 100 mesh, Sunresin New Materials Co., Ltd., Xi’an, China). For property comparison and the CEDI experiment, commercial heterogeneous IEMs, IONAC MC-3470, and MA-3475 were used, supplied by Lanxess Sybron Chem. Inc. (Birmingham, NJ, USA). The cation-exchange resin (CER) and anion-exchange resin (AER) employed in the CEDI experiments were provided by Samyang Corp. (Seoul, Republic of Korea).

### 2.2. Fabrication of Heterogeneous AEMs

**Figure 2:**
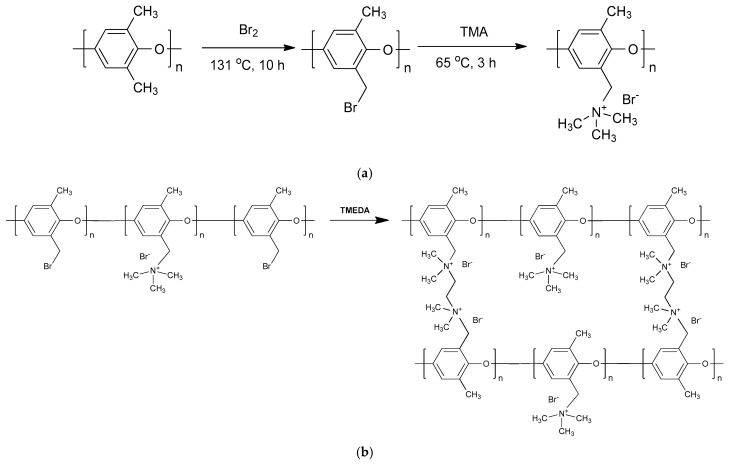
(**a**) Reaction schemes for preparing BPPO and QPPO, and (**b**) crosslinking mechanism of QPPO using diamine.

To prepare the ionomer binder, BPPO was first synthesized by reacting PPO with bromine (Br_2_). PPO was dissolved in chlorobenzene at a concentration of 8 wt%, and the bromination reaction was conducted by slowly adding a 20 wt% Br_2_ solution in chlorobenzene, dropwise, in a nitrogen atmosphere while heating at 131 °C for 10 h. The molar ratio of PPO (repeating unit) to Br_2_ was maintained at 1:1. Following the reaction, the BPPO solution was precipitated in methanol, washed several times with methanol, and dried in a vacuum oven at 60 °C for over 12 h [29,30]. The resulting BPPO was then dissolved in NMP (a co-solvent) at 15 wt%, and TMA (45 wt% in water) was added based on the molar ratio of TMA to BPPO, reacting at 65 °C for 3 h. QPPO was subsequently prepared by adjusting the molar ratio of TMA to BPPO (repeating unit) to 0.1, 0.2, 0.3, and 0.4. After the quaternization reaction, the QPPO solution was precipitated in distilled water (DW), washed with DW, and dried in a 50 °C vacuum oven for over 10 h. The reaction schemes for BPPO and QPPO preparation are shown in Figure 2a. To create a free-standing membrane, QPPO was dissolved in NMP at 25 wt% and cast using a solution casting method. The cast membrane was then dried in a vacuum oven at 50 °C for more than 10 h to produce a QPPO membrane approximately 35 μm thick in its dry state.

Nanofibers, intended as additives for the heterogeneous membranes, were prepared using QPPO with a TMA/BPPO molar ratio of 0.35. This was conducted through electrospinning using a commercial machine (ESR200R2, NanoNC, Seoul, Republic of Korea). The QPPO solution was loaded into a syringe and electrospun by applying a 25 kV electric field at a flow rate of 0.6 mL/h. The resulting nanofibers were crosslinked by immersing them in a 20 wt% TMEDA aqueous solution for 2 h. This crosslinking occurs due to the chemical bonds formed between the amine groups at both ends of TMEDA and the brominated moieties in QPPO, as illustrated in Figure 2b. After crosslinking, the QPPO nanofibers were washed multiple times with DW, vacuum filtered, and dried in a vacuum oven at 40 °C for over 10 h. The dried nanofibers were then finely ground in a mortar and added to the casting solution for the fabrication of heterogeneous membranes.

**Figure 3:**
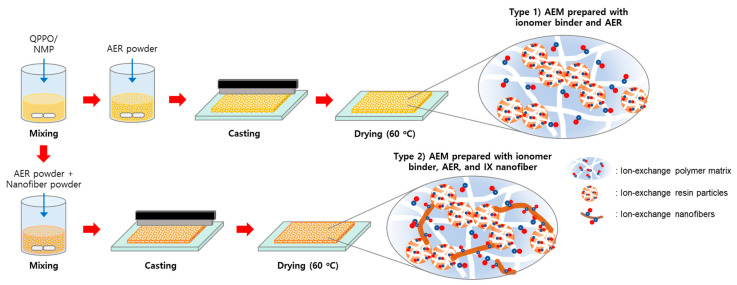
Heterogeneous membrane fabrication scheme.

To prepare the heterogeneous AEMs, we formulated mixture solutions with various compositions. These solutions were then cast onto a glass substrate to form films with a thickness of 240 μm. The films were vacuum-dried at 60 °C for over 6 h. NMP was used as the solvent in preparing the casting solutions, and the quantities of ionomer binders (prepared at different TMA/BPPO ratios) and QPPO nanofibers were carefully adjusted. The membrane fabrication process is schematically illustrated in Figure 3.

### 2.3. Membrane Characterizations

The chemical structure of the ionomer binder used in producing the heterogeneous membranes was confirmed using Fourier transform infrared spectroscopy (FT-IR, FT-IR-4700, Jasco, Tokyo, Japan). The samples for this analysis were prepared as free-standing films. The measurement of each sample involved 16 scans at a resolution of 4 cm^−1^. The analysis of the data was conducted using graphs representing the average of the collected data.

The morphological characteristics of the prepared membranes were examined using a scanning electron microscope (SEM, Cube II, EmCrafts Co., Ltd., Hanam, Republic of Korea). Through the analysis of SEM images, both the surface and cross-sectional shapes of the membrane samples were observed. Additionally, the heterogeneity resulting from the incorporation of IER powder into the membranes was also confirmed through this analysis.

To measure the water uptake (WU) of the membrane samples, we first cut the membranes into pieces measuring 2 cm × 2 cm. These pieces were then immersed in DW at room temperature. After the immersion, we carefully removed the excess water from the surface of each sample using filter paper. The wet weight of each sample (*W_wet_*, g) was then measured. Subsequently, the samples were dried in an oven at 60 °C for over 10 h, allowing us to obtain their dry weight (*W_dry_*, g). The WU value was calculated using these measured weights, substituted into Equation (1) [31]:

(1) $$\mathrm{WU}=\frac{W_{wet}-W_{dry}}{W_{dry}}\times 100\;\left[\%\right].$$

The IEC of the prepared AEMs was measured using the Mohr method. Initially, the membrane samples were immersed in a 0.5 M NaCl solution. Once equilibrium was reached between the solution and the membrane, the membrane sample was washed several times with DW and then immersed in a 0.25 M Na_2_SO_4_ solution for over 6 h. To carry out the titration, 4–5 drops of a 1 wt% K_2_CrO_4_ indicator were added to this solution. The solution was then titrated with 0.01 N AgNO_3_ to quantitatively analyze the amount of Cl^−^ ions that had been substituted in the membrane. Finally, the IEC values were calculated using the following equation [32,33,34]:

(2) $$\mathrm{IEC}=\frac{C\cdot V_{s}}{W_{dry}}\;\left[\frac{\mathrm{meq}.}{g_{\mathrm{dry}\;\mathrm{memb}}}\right]$$

where *C* is the normal concentration of the titrant solution (meq./L), *V_s_* is the solution volume (L), and *W_dry_* is the weight of the dry membrane (g).

The electrical resistance (ER) of the membranes was measured in a 0.5 M NaCl solution using a lab-made 2-point probe clip cell and an impedance analyzer (SP-150, Bio-Logic Science Instruments, Seyssinet-Pariset, France) [35]. The ER values were determined using the following equation [32,33]:

(3) $$\mathrm{ER}=\left(R_{1}-R_{2}\right)\times A\;\left[\Omega \;{\mathrm{cm}}^{2}\right]$$

where *R*_1_ is the resistance of electrolyte and membrane (Ω), *R*_2_ is the resistance of electrolyte only (Ω), and *A* is the membrane area (cm^2^).

The ion conductivity (*σ*) of the membranes was calculated using the following equation, which takes into account the previously measured membrane resistance, as well as the membrane’s area and thickness:

(4) $$\sigma =\frac{L}{R\times A}\;\left[\mathrm{mS}/\mathrm{cm}\right]$$

where *L* is the thickness (cm), *R* is the resistance (Ω), and *A* is the area (cm^2^) of the membrane sample.

The ion transport number (TN, *t*___), which reflects the selective permeability of an IEM, was measured using the electromotive force (emf) method in a two-compartment diffusion cell. The electrolyte solutions in both compartments were adjusted to have a fivefold difference in concentration. A pair of Ag/AgCl electrodes were connected to a digital multimeter to measure the potential difference across the membrane. From these measurements, the TN value was calculated using the following equation [33,36,37]:

(5) $$E_{m}=\frac{RT}{F}(1-2t_{\_})\ln\frac{C_{L}}{C_{H}}$$

where *E_m_* is the measured cell potential (V), *R* is the ideal gas constant (8.31 J/K mol), *T* is the absolute temperature (K), *F* is the Faraday constant (96,500 C/mol), and *C_L_* (1 mM) and *C_H_* (5 mM) are the concentrations of NaCl solution.

The tensile strength of the membrane was measured in accordance with international standards, specifically following the ASTM method D-882-79. For this measurement, wet membrane samples were cut into pieces measuring 7 cm × 1 cm. These samples were then tested using a universal testing machine (34SC-1, Instron, Norwood, MA, USA).

The contact angle of the dry membrane was measured using a contact angle analyzer (Phoenix 150, SEO, Suwon, Republic of Korea).

### 2.4. CEDI Performance Test

**Figure 4:**
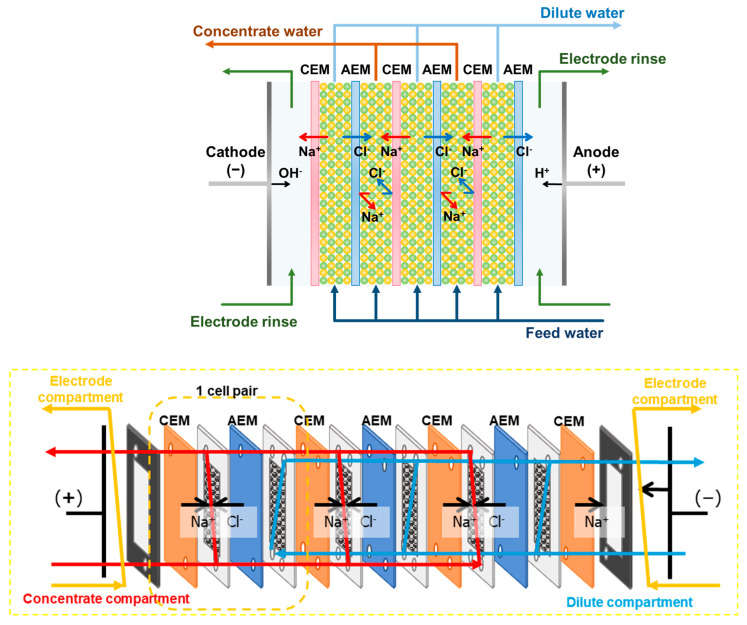
Schematic drawings of CEDI module configuration and operation principles.

CEDI tests were conducted to assess the performance of the fabricated membranes. The configuration and operating principles of the CEDI module used in this experiment are depicted in Figure 4. The module was comprised of four CEMs and three AEMs, each with an effective area of 15 cm^2^, and was equipped with a pair of Pt-plated Ti electrodes. The gaskets, determining the volume of the compartments in the module, had thicknesses of 1 mm for the concentrate compartment and 3 mm for the dilute compartment. These compartments were filled with 1.5 cm^3^ and 4.5 cm^3^ of mixed ion exchange resin (IER) in a 1:1 volume ratio of CER to AER, respectively. A 200 mL solution of 0.005 M NaCl was circulated through the module at a flow rate of 50 mL/min as influent. The electrode compartment circulated 250 mL of 0.01 M Na_2_SO_4_ at 30 mL/min. A DC power supply (MK6005P, MK Power, Seoul, Republic of Korea) was used to apply 10 V to the electrode in constant voltage (CV) mode for 1 h. During the experiments, the pH and conductivity of the solutions in the concentrate and dilute compartments were measured every 10 min using an Orion Star™ A215 pH/conductivity benchtop multiparameter meter (Thermo Fisher Scientific Inc., Waltham, MA, USA). Samples were also taken for ion concentration analysis. All of the experiments were conducted at room temperature.

The ion removal rate (*R*), which is a key parameter for evaluating the performance of the CEDI process, was calculated using the following equation [38]:

(6) $$R=\frac{C_{F}-C_{D}}{C_{F}}\times 100\;\left[\%\right]$$

where *C_F_* is the feed solution conductivity (μS/cm) and *C_D_* is the dilute solution conductivity (μS/cm).

Energy consumption (*η*) can be defined as the energy required to transport ions from the feed to the concentration compartment and is determined using the following equation [39]:

(7) $$\eta =\int_{0}^{t}VI\;dt\;\left[\mathrm{Wh}\right]$$

where *V* is the cell voltage (V), *I* is the current (A), and *t* is the time (h).

## 3. Results and Discussion

**Figure 5:**
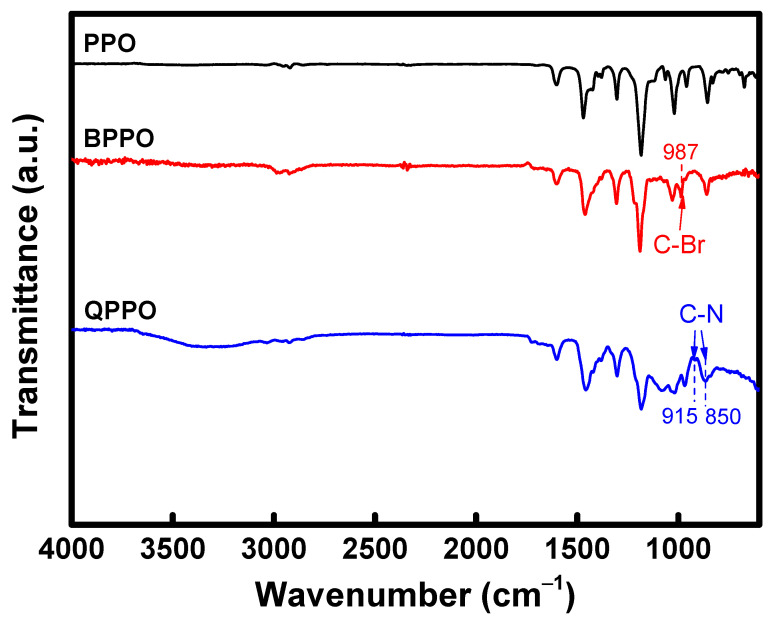
FT-IR spectra of PPO, BPPO, and QPPO.

The FT-IR spectra, used to confirm the chemical structure of the prepared QPPO, are displayed in Figure 5. Initially, the presence of a C-Br stretching peak at 987 cm^−1^ in the spectrum of BPPO indicates that the bromination reaction occurred successfully, achieving chemical modification of PPO [40,41]. The degree of bromination in the prepared BPPO was confirmed to be approximately 55% through NMR analysis. However, in the QPPO spectrum, the disappearance of the C-Br absorption band suggests that bromine was ionized, leading to the quaternization reaction with the tertiary amine, TMA. Additionally, a broad absorption band around 3500 cm^−1^ in the QPPO spectrum was identified as the vibration of N-H stretching and O-H stretching in the quaternary ammonium groups [42,43], implying an increase in the polymer’s hydrophilicity due to the introduction of these groups. Furthermore, the appearance of peaks at 850 cm^−1^ and 915 cm^−1^ following the reaction with TMA, are indicative of C-N stretching in the quaternary ammonium groups, confirming the successful preparation of QPPO [42,43,44].

Basic characterizations were performed to determine the optimal conditions for using QPPO as a binder in heterogeneous membranes, with the results summarized in Table 1. These results indicate that an increase in the amount of TMA added leads to higher IEC and WU values. Furthermore, as the TMA/BPPO molar ratio increased, the number of quaternary ammonium groups introduced into QPPO also rose, resulting in reduced electrical resistance and enhanced ion conductivity of the membrane [45]. Consequently, QPPO with IEC values ranging from 0.26 to 1.87 meq./g was successfully prepared by adjusting the TMA/BPPO molar ratio. Notably, for QPPO with a TMA/BPPO molar ratio of 0.4, which exhibited an IEC of 1.87 meq./g (comparable to that of homogeneous IEMs) and a WU close to 30%, it was anticipated that the mechanical properties of the membrane could be compromised if used as a binder. Therefore, this composition was excluded from the binder formulation for heterogeneous membrane fabrication.

**Figure 6:**
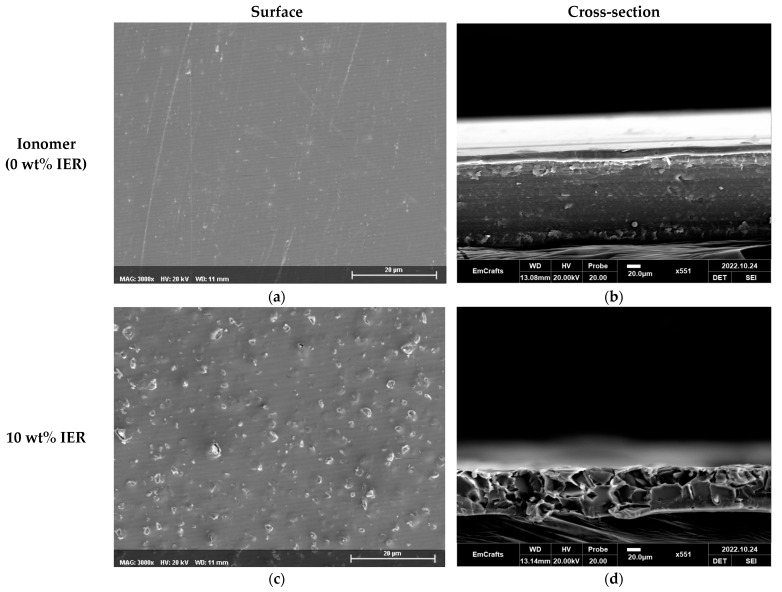

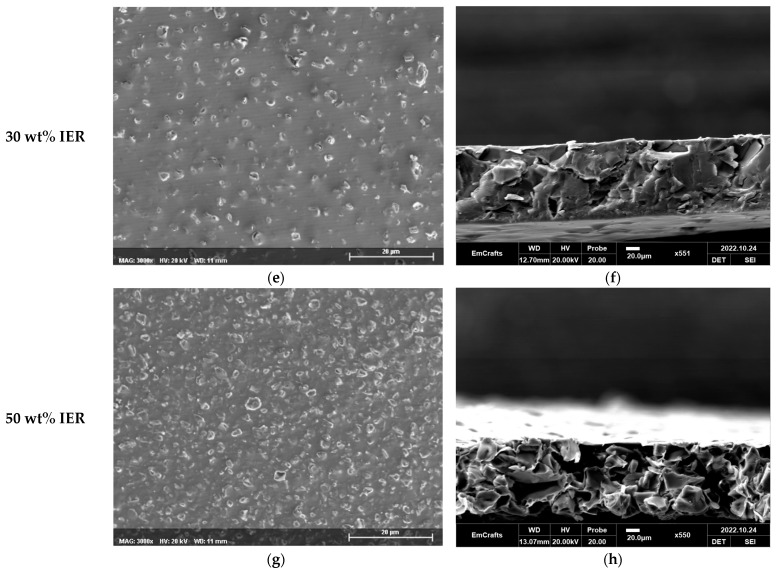
SEM images of the surface (left, magnification of 3000×) and cross-section (right, magnification of 550×) of the fabricated AEMs ((**a**,**b**), (**c**,**d**), (**e**,**f**) and (**g**,**h**) represent the membranes with IER content of 0%, 10%, 30%, and 50%, respectively).

SEM images displaying the surface and cross-section, which reveal the morphological characteristics of the fabricated heterogeneous membranes, are presented in Figure 6. Specifically, Figure 6a,c,e,g show SEM images at 3000× magnification of the surfaces of AEMs prepared with 0%, 10%, 30%, and 50% IER contents, respectively. In addition, 550× magnified cross-sectional images of AEMs fabricated with 0%, 10%, 30%, and 50% IER powders are depicted in Figure 6b,d,f,h, respectively. It is observed that as the IER powder content in the casting solution for membrane fabrication increases, the quantity of resin particles visible on both the surface and cross-section correspondingly increases. These particles are shown to be uniformly dispersed throughout the membranes.

**Figure 7:**
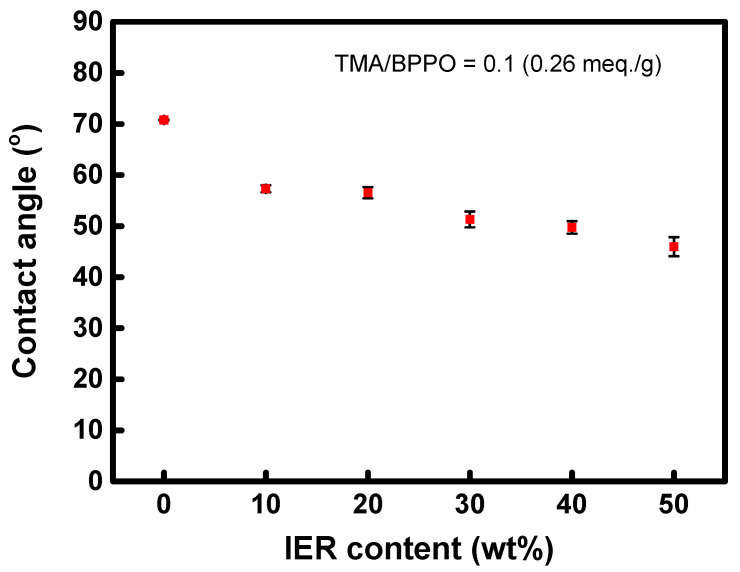
Surface contact angles of heterogeneous membranes prepared with different IER contents.

The surface contact angle results of the heterogeneous AEMs prepared with a TMA/BPPO molar ratio of 0.1, as a function of the IER content, are illustrated in Figure 7. As the hydrophilic IER content increases, the contact angle on the membrane surface tends to decrease gradually, indicating an elevation in the membrane’s surface hydrophilicity. This increase in surface hydrophilicity is beneficial as it can reduce ion transfer resistance at the interface and enhance the efficiency of water splitting. In comparison, the contact angle of the commercial membrane, IONAC MA-3475, was measured at 58.06°. This finding suggests that the fabricated membranes exhibit higher surface hydrophilicity than the commercial membrane under all tested fabrication conditions.

**Figure 8:**
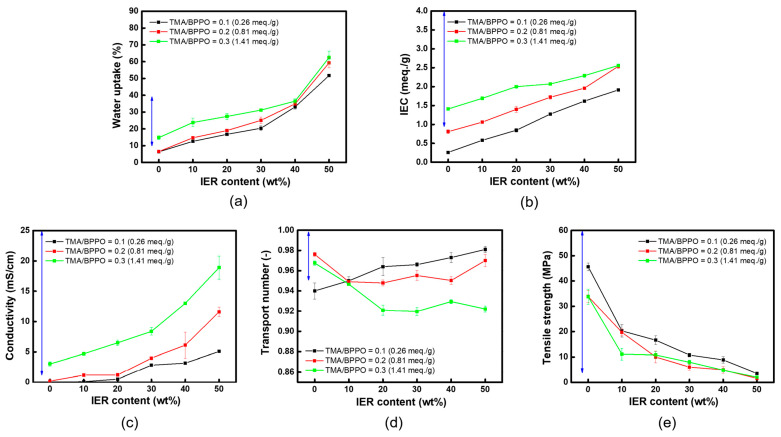
(**a**) Water uptake, (**b**) ion-exchange capacity, (**c**) conductivity, (**d**) tensile strength, and (**e**) transport number of heterogeneous membranes prepared with different IER contents (the blue double arrow indicates the appropriate range of membrane performance parameters determined based on the literature review).

The WU and IEC of the heterogeneous AEMs prepared using the QPPO binder are depicted in Figure 8a and Figure 8b, respectively. Similar to the scenarios where an inert polymer binder is used, both WU and IEC values were observed to increase with the rising content of IER powder in the heterogeneous membranes. This increase in IER powder content results in a greater number of hydrophilic ion-exchange groups within the membrane. Consequently, due to the heterogeneous interface between the IER particles and the binder, more pronounced swelling occurs, leading to an elevation in WU [5,45].

The ion conductivity data for the prepared heterogeneous membranes are presented in Figure 8c. It is observed that the ion conductivity of the membranes increases with the rising IEC values. However, it is important to note that an excessive increase in ion conductivity might result from a decrease in permselectivity, which allows both counter-ions and co-ions to pass through the membrane. Therefore, it is necessary to confirm this by measuring the ion transport number, as a high ion conductivity coupled with low permselectivity could be less desirable for certain applications [46].

The variations in the TNs of the heterogeneous AEMs, correlating with the IEC of the QPPO binder and the content of IER powder, are illustrated in Figure 8d. Specifically, for the heterogeneous membrane prepared using QPPO with a TMA/BPPO molar ratio of 0.1 (IEC = 0.26 meq./g) as a binder, an increase in TNs was observed as the IER powder content increased. This trend is interpreted as a consequence of heightened electrostatic repulsion. Conversely, when using a QPPO binder with a higher IEC (i.e., TMA/BPPO = 0.2 and 0.3), the TNs initially decreased with increasing IER powder content, then stabilized or showed a slight increase. This behavior suggests that a high IEC in the QPPO binder, coupled with the addition of hydrophilic IER powder, can cause significant membrane swelling and potentially reduce permselectivity. However, as the degree of swelling increases and the electrostatic repulsion improves—owing to a rise in the number of ion exchange groups—the TNs might stabilize or slightly increase after an initial decrease [46,47,48].

In addition, to verify the impact of the IEC of the QPPO binder and the IER powder content on the mechanical properties of the heterogeneous AEMs, the tensile strength of the membranes was measured. The results are presented in Figure 8e. It was observed that an increase in the IEC of the QPPO binder led to a slight decrease in tensile strength. This decrease is attributed to a deterioration in the mechanical properties of the membranes, likely due to an increase in the swelling ratio [5]. Conversely, as the content of IER powder increased, the tensile strength of the membranes showed a significant decrease. This trend is interpreted as a consequence of a substantial increase in the swelling ratio, driven by the higher content of hydrophilic IER powder and the presence of a weaker physical bond at the binder-IER particle interface [48]. These findings correspond well with the WU data illustrated in Figure 8a, indicating a consistent trend across different measurements.

The optimal fabrication conditions for heterogeneous membranes, ascertained through literature reviews and experimental findings, are summarized in Table 2. The ideal ion conductivity was established as exceeding 2 mS/cm, based on the ion conductivity of commercial heterogeneous IEMs [44]. Generally, for IEMs, lower WU correlates with better mechanical properties, yet a minimum of 10% WU is necessary due to the presence of hydrophilic fixed charge groups. However, exceeding approximately 40% in WU may lead to compromised permselectivity and mechanical strength. Consequently, the optimal WU range was set between 10% and 40%. Since an IEC of 1.0 meq./g or higher is essential to maintain minimum permselectivity and ion conductivity, akin to homogeneous IEMs, this value was selected as the appropriate lower limit. The lower threshold for the TN was determined based on the intermediate values typical of both homogeneous and heterogeneous membranes. Furthermore, considering the tensile strength (approximately 3.5 MPa) of commercial heterogeneous IEMs used in CEDI modules without reinforcement, a desirable tensile strength range of above 3 MPa was identified.

**Figure 9:**
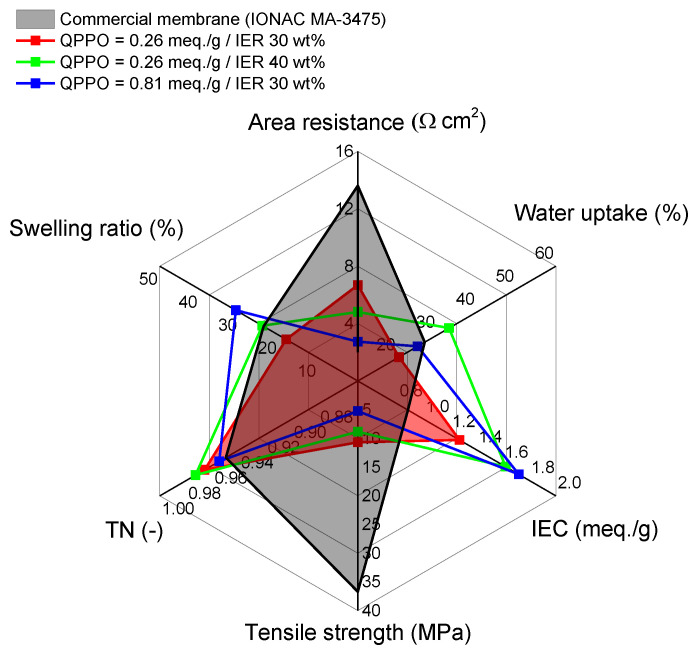
**A** spider chart showing the values of key performance parameters of heterogeneous AEMs.

As a result, three different compositions of heterogeneous AEMs, fabricated using a QPPO binder, were found to meet all the optimal membrane fabrication conditions mentioned above. The performance parameters of these membranes were compared with those of IONAC MA-3475, a commercial membrane. This comparison is visually represented as a spider chart in Figure 9. Overall, the properties of the fabricated membranes were found to be superior to those of the commercial IONAC membrane, particularly in terms of electrical resistance. However, regarding tensile strength, while the results surpassed those of conventional heterogeneous membranes without reinforcing materials, they were not as high as those of the IONAC membrane, which is fabricated with a reinforcing material. In conclusion, a TMA/BPPO molar ratio of 0.1 and an IER powder content of 30 wt%, which yielded the highest tensile strength among the tested fabrication conditions, were identified as the optimal conditions for producing the heterogeneous AEM.

**Figure 10:**
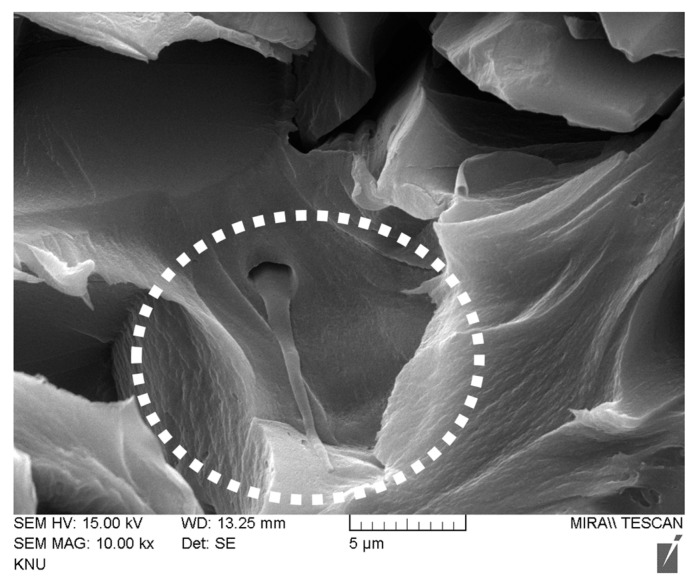
Cross-sectional SEM image of heterogeneous AEM prepared with ionomer binder and IX nanofiber additive (dashed circle indicates IX nanofiber).

**Figure 11:**
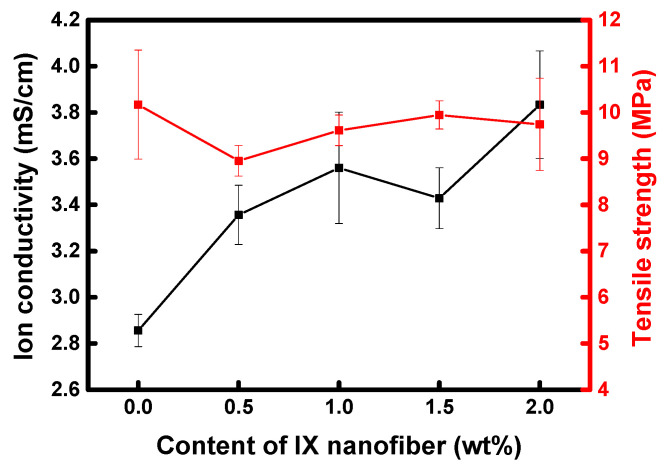
Ion conductivity and tensile strength of heterogeneous AEMs according to IX nanofiber content.

Additionally, to balance the objectives of maintaining tensile strength and increasing ion conductivity, a heterogeneous membrane was fabricated by combining crosslinked QPPO nanofiber powder with an ionomer binder. The FE-SEM image, as shown in Figure 10, confirms the incorporation of QPPO nanofibers into the membrane. These added nanofibers were anticipated to enhance ion conduction connectivity between IER particles while also preserving the physical stability of the membrane. Generally, an increase in IEC or the addition of IER particles to IEMs tends to boost ion conductivity, but at the cost of diminished mechanical properties. However, as depicted in Figure 11, with the increased content of crosslinked QPPO nanofibers, not only is the ion conductivity of the heterogeneous membrane significantly enhanced, but the tensile strength is also maintained at a stable level.

**Figure 12:**
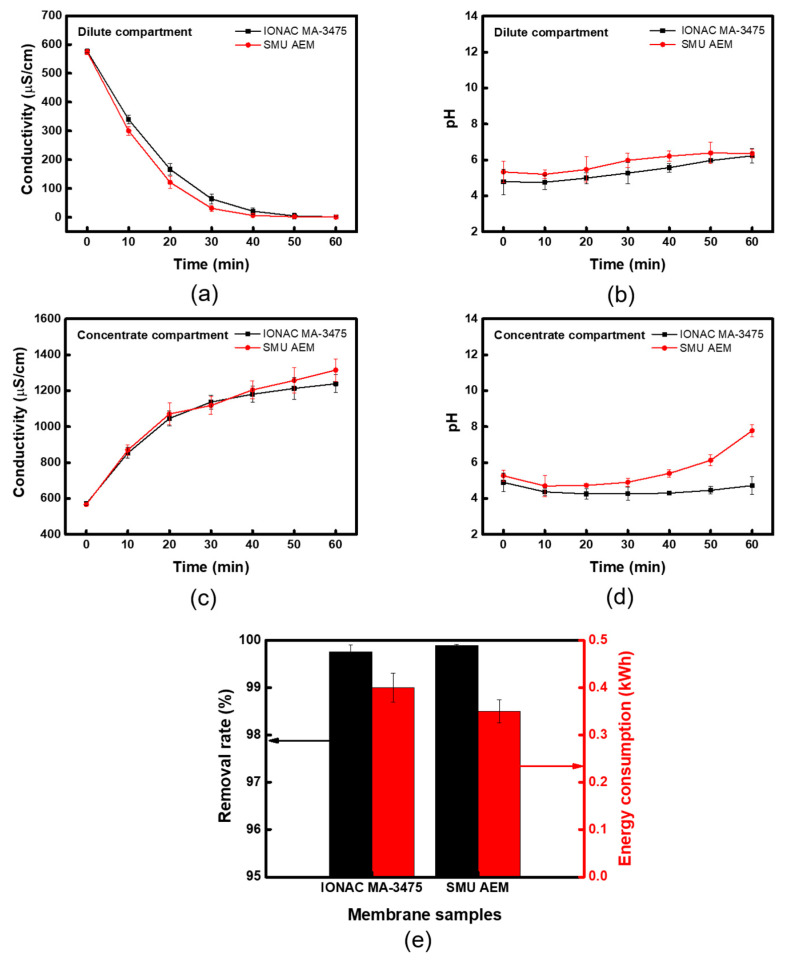
CEDI performances of commercial and prepared heterogeneous membranes: (**a**) conductivity and (**b**) pH of the dilute compartment, (**c**) conductivity and (**d**) pH of the concentrate compartment, and (**e**) removal rate and energy consumption.

Figure 12 presents the results of the CEDI experiment comparing a commercial IONAC membrane with a specially fabricated heterogeneous membrane (SMU), prepared under the optimal conditions (i.e., TMA/BPPO molar ratio = 0.1, IER powder content = 30 wt%, and nanofiber content = 2 wt%). The results demonstrated a more pronounced change in ion conductivity in both the dilute and concentrate compartments when using the SMU membrane compared to the IONAC membrane. This superior performance is primarily attributed to the lower electrical resistance of the SMU membranes. Additionally, the relatively stable maintenance of solution pH during desalination indicates effective regeneration of the IER bed. When ion concentration in the dilute compartment drops significantly, enhanced water-splitting at the AEM, resulting in increased OH-ion transport, can cause a rise in pH in the concentrate compartment. With the SMU membrane, ions were more rapidly removed from the dilute compartment, leading to accelerated water-splitting reactions. Consequently, the heterogeneous membrane fabricated with the ionomer binder demonstrated excellent salt removal efficiency (99.86%) and low energy consumption (0.35 kWh), outperforming the commercial membrane (which showed 99.76% salt removal efficiency and 0.4 kWh energy consumption). These superior results are interpreted as arising from the significantly lower electrical resistance and improved permselectivity of the SMU membrane compared to the commercial membrane.

## 4. Conclusions

In this study, we derived the optimal fabrication conditions for a heterogeneous AEM with exceptional performance for application in CEDI. To achieve this, QPPO containing quaternary ammonium groups was selected as the binder for preparing the heterogeneous membranes. We adjusted the IEC of the QPPO binder and the content of IER powder in various configurations. The resulting membranes underwent systematic analysis to evaluate their properties. It was found that the optimal IER powder content is dependent on the IEC of the QPPO binder. Additionally, we established appropriate ranges for crucial performance parameters of the membrane, including ion conductivity, WU, IEC, tensile strength, and TN. Membrane fabrication conditions meeting all these criteria were identified. Ultimately, the ideal membrane fabrication conditions, surpassing the performance of commercial membranes, were determined to be an IEC (QPPO binder) of 0.26 meq./g and an IER content of 30 wt%. This suggests that the ionomer binder should have a significantly lower IEC than a homogeneous IEM, and the IER powder content should be lower than that in conventional heterogeneous IEMs. Additionally, crosslinked QPPO nanofibers, prepared via electrospinning, were incorporated as an additive to enhance ion conductivity while preserving the membranes’ mechanical strength. When the optimally fabricated heterogeneous AEM was applied to CEDI for ultrapure water production, it demonstrated superior desalination efficiency (99.86%) and lower energy consumption (0.35 kWh) compared to commercial membranes (99.76% and 0.4 kWh, respectively). The findings of this study are anticipated to address the limitations of conventional heterogeneous IEMs and significantly enhance their performance.

## Figures and Tables

**Table 1:** The basic properties of prepared QPPO membranes.

QPPO (TMA/BPPO, Mole Ratio)	Thickness (μm)	WU (%)	IEC (meq./g)	ER (Ω·cm^2^)	Conductivity (mS/cm)
0.1	68.2	6.34	0.26	573	0.01
0.2	53.8	6.50	0.81	22.0	0.22
0.3	46.8	14.8	1.41	3.74	3.06
0.4	45.9	27.9	1.87	1.05	4.38

**Table 2:** Criteria for optimal fabrication conditions of heterogeneous IEMs.

Properties	Conductivity (mS/cm)	WU (%)	IEC (meq./g)	Tensile Strength (MPa)	Transport Number (−)
Criteria	>2	10~40	>1	>3	>0.95

## Data Availability

Data are contained within the article.

## Abbreviations

AEM	anion-exchange membrane
AER	anion-exchange resin
BPM	bipolar membrane
BPPO	brominated PPO
CEDI	continuous electrodeionization
CEM	cation-exchange membrane
CER	cation-exchange resin
CV	constant voltage
DC	direct current
DW	distilled water
ED	electrodialysis
ER	electrical resistance
FT-IR	Fourier transform infrared spectroscopy
IEC	ion-exchange capacity
IEM	ion-exchange membrane
IER	ion-exchange resin
NMP	1-methyl-2-pyrrolidone
PEG	polyethylene glycol
PPO	poly(2,6-dimethyl-1,4-phenylene oxide)
QPPO	quaternized PPO
SEM	scanning electron microscope
TMA	trimethyl amine
TMEDA	*N,N,N′,N′*-tetramethylethylenediamine
TN	transport number
WU	water uptake
